# Associations of the Healthy Beverage Index and the risk of colorectal cancer: a case-control study

**DOI:** 10.3389/fnut.2025.1595246

**Published:** 2025-08-08

**Authors:** Amr Ali Mohamed Abdelgawwad El-Sehrawy, Mundher Kadem, Irfan Ahmad, Ahmed Hjazi

**Affiliations:** ^1^Department of Internal Medicine, Diabetes, Endocrinology and Metabolism, Mansoura University, Mansoura, Egypt; ^2^Department of Medical Analysis, Medical Laboratory Technique College, The Islamic University of Al Diwaniyah, Al Diwaniyah, Iraq; ^3^Department of Clinical Laboratory Sciences, College of Applied Medical Sciences, King Khalid University, Abha, Saudi Arabia; ^4^Department of Medical Laboratory, College of Applied Medical Sciences, Prince Sattam Bin Abdulaziz University, Al-Kharj, Saudi Arabia

**Keywords:** Healthy Beverage Index, HBI, colorectal cancer, dietary, case-control

## Abstract

**Background:**

Colorectal cancer (CRC) remains one of the leading causes of cancer-related morbidity and mortality worldwide. Emerging evidence suggests that dietary patterns can significantly influence CRC risk, with beverages playing a critical role. The Healthy Beverage Index (HBI) is a tool to assess the healthfulness of beverage consumption, yet its relationship with colorectal cancer risk has not been extensively studied.

**Methods:**

A total of 250 participants diagnosed with colorectal cancer and 250 age- and sex-matched control subjects were recruited for the study. Beverage intake was assessed using a validated dietary questionnaire, and HBI scores were calculated to reflect the quality of beverage consumption. Logistic regression analyses were performed to examine the association between HBI scores and colorectal cancer risk, controlling for potential confounders such as energy intake, physical activity, family history of cancer, and other lifestyle factors were assessed.

**Results:**

The case group had an average age of 48.91 years and BMI of 29.61, while the control group averaged 47.13 years and 29.07 BMI. CRC patients had a higher waist circumference (*p* < 0.05) and lower vitamin D intake and HBI scores than controls (*p* < 0.05). Those in the highest HBI quartile consumed more nutrients compared to the lowest (*p* < 0.05). Higher HBI scores correlated with increased physical activity. The highest HBI quartile significantly reduced CRC odds (OR: 0.28; 95% CI: 0.19–0.51), remaining significant after adjustments (OR: 0.43; 95% CI: 0.25–0.76).

**Conclusion:**

The HBI is inversely associated with the risk of colorectal cancer, suggesting that improvements in beverage choices may serve as an effective dietary strategy for CRC prevention. These results underscore the critical role of beverage consumption in dietary assessments and cancer risk management, warranting further examination in prospective studies.

## Introduction

Colorectal cancer (CRC) is a major global health problem, with a high incidence and the second highest rate of cancer-related deaths (1). In 2020, the World Health Organization estimated 1.93 million new CRC cases and 935,000 deaths (2). Rising trends in low-income countries are compounded by aging populations and the increased adoption of Western lifestyles, creating an urgent need for effective prevention and management strategies. Diet, obesity, physical inactivity, smoking, and excessive alcohol intake are modifiable risk factors for CRC. High consumption of red and processed meats, coupled with a low-fiber diet and a sedentary lifestyle, significantly increases the risk (3). Preventative strategies refer to the lifestyle modifications to prevent these risks and hence reduce overall incidence (4). Dietary interventions have great potential for CRC prevention. Increasing fiber intake, reducing red and processed meat consumption, and increasing the intake of fruits and vegetables can significantly reduce risk. However, these dietary shifts, combined with regular physical activity, form the cornerstone of effective CRC prevention strategies (5).

A novel assessment tool for evaluating the quality of beverage intake in relation to chronic disease prevention is the Healthy Beverage Index (HBI). This index measures consumption patterns based on criteria such as sugar content, the presence of beneficial nutrients, and overall caloric contribution (6). Selecting water, unsweetened teas, and nutrient-dense drinks, such as certain juices, can help optimize HBI scores, improve overall dietary quality, and decrease disease risk (7).

A high intake of sugary beverages appears to increase inflammation and insulin resistance, which may raise colorectal cancer risk, according to studies (8). Regular consumption of coffee, which is rich in antioxidants and polyphenols, has been linked to a lower risk, most likely due to its anti-inflammatory properties (9). The antiangiogenic and antiproliferative mechanisms of green tea catechins also protect against colorectal cancer. A link between alcohol and colorectal cancer has been established, particularly with excessive consumption, possibly through the production of acetaldehyde and the impairment of folate metabolism (10).

Despite growing evidence on the impact of beverage consumption on colorectal cancer risk, few studies have comprehensively evaluated this relationship using standardized measures such as the HBI. This case-control study aims to investigate the association between HBI and the risk of colorectal cancer.

## Method

### Study population

We conducted a comprehensive hospital-based case-control study at King Khalid Hospital. Cases included patients aged 30–79 years with pathologically confirmed colorectal cancer (CRC), diagnosed within 3 months prior to the interview. Patients with a history of other cancers or previous adenomatous polyps were excluded. Additionally, patients diagnosed with adenomatous colorectal polyps via colonoscopy and histological confirmation were included as a separate group.

Control subjects were randomly selected from individuals admitted to the same hospital during the same period. Controls were frequency-matched to cases by age (±10 years) and sex. Inclusion criteria for controls were admission for acute or minor non-neoplastic conditions unrelated to diet-related chronic illnesses (such as fractures, minor infections, or elective surgeries). Controls with any history of cancer, adenomatous polyps, or chronic diseases related to diet (e.g., diabetes, cardiovascular disease, chronic kidney disease) were excluded to minimize confounding.

Out of 516 participants initially recruited (259 controls, 257 CRC cases), 16 individuals (seven controls and nine cases) were excluded due to incomplete food frequency questionnaires or total energy intake values outside ±3 standard deviations from the mean. The final sample included 250 cases and 250 controls.

The research protocol was reviewed and approved by the Ethics Committee of King Khalid University. All participants provided written informed consent prior to their inclusion in the study. This study was approved by the Ethics Committee of King Khalid University (approval code: ECM#2023-11-137).

### Dietary intake assessment

The dietary intake of the participants was assessed through the utilization of a validated semi-quantitative food frequency questionnaire, which comprised a total of 152 distinct food items (11). Participants were instructed to report their average consumption of various food items over the course of the previous year by selecting one of several predefined options. These options included: never or less than once a month, three to four times a month, once a week, two to four times a week, five to six times a week, once a day, two to three times a day, four to five times a day, or six times or more each day. Finally, the calculated daily intake was input into Nutritionist IV software to determine total energy and nutrient intake.

### Assessment of anthropometric variables and physical activity

Standardized methodologies were employed to systematically gather anthropometric data from the participants involved in the study. The weights of the patients were accurately assessed while they were attired in comfortable clothing and without any footwear, utilizing a Seca digital scale, which is a product manufactured in Germany and is known for its precision, capable of measuring weight with an accuracy of 100 g. In addition, the measurement of standing height was conducted using a tape measure, ensuring that the participants were barefoot during this process. To calculate the body mass index (BMI), the weight of the individual in kilograms was divided by the square of their height measured in meters squared (m^2^). Furthermore, waist circumference (WC) was measured at its narrowest point using a non-elastic tape, ensuring that no pressure was exerted on the surface of the body during the measurement process.

We employed a reliable abbreviated version of the International Physical Activity Questionnaire (short IPAQ) to assess the physical activity levels of participants. The calculation of physical activity in MET-minutes per week was performed as follows: Walking MET-minutes/week = 3.3 × Walking minutes × Walking days. Moderate MET-minutes/week = 4.0 × Minutes of moderate-intensity activity × Days of moderate activity. Vigorous MET-minutes/week = 8.0 × Minutes of vigorous-intensity activity × Days of vigorous activity. The total physical activity in MET-minutes/week was determined by summing the scores of Walking, Moderate, and Vigorous MET-minutes/week. Additionally, we secured informed written consent from all participants.

### Calculation of Healthy Beverage Index (HBI)

The HBI was developed by Duffey and Davy (12), as referenced in their work. This index serves a purpose similar to that of the Healthy Eating Index, as it can be utilized to assess the overall quality of beverage consumption and to detect any alterations in drinking habits over time. It is important to note that health-related changes are often associated with specific consumption patterns. In the framework of the beverage guidance system, all items that were classified as beverages were categorized into eight distinct types. These categories include 100% fruit juice, plain water, unsweetened varieties of coffee and tea, low-fat milk, diet beverages—which encompass caffeine-free coffee and tea as well as other drinks that are artificially sweetened—alcoholic beverages like beer, wine, and spirits, and finally, full-fat milk. Eight distinct categories of beverages were consumed by individuals, which included fruit juices, sweetened coffee and tea, as well as carbonated soft drinks. A higher score indicates greater adherence to drinking standards and healthier drinking practices, with the final HBI score ranging from 0 to 100 (12). Since our study’s target demographic did not consume diet beverages (rated between 0 and 5) or alcoholic drinks (scoring between 0 and 5), the highest possible final HBI score was 90. The focus of this research was to examine compliance with healthy beverage consumption guidelines rather than total fluid intake; therefore, liquids consumed during meals (like soup) were excluded.

### Statistical analysis

Statistical analyses were conducted using SPSS software (version 26.0; SPSS Inc., Chicago IL). The Shapiro–Wilk tests evaluated the normal distribution of the variables. Baseline characteristics and dietary intakes for quantitative factors were expressed as mean ± standard deviation (SD), while qualitative variables were reported as counts and percentages. We compared the data between two groups using independent sample *T*-Tests or, when appropriate, the non-parametric alternative (Mann–Whitney test) for continuous variables, and chi-squared tests for categorical variables. A conditional logistic regression model was employed to calculate odds ratios (ORs) and 95% confidence intervals (CIs), adjusting for various covariates in a separate model. In all analyses, a significance level of *p* < 0.05 was established.

## Results

The average (SD) age and BMI of the case groups were 48.91 (10.46) years and 29.61 (4.55) kg/m^2^, respectively, whereas the average age and BMI of the control groups were 47.13 (10.08) years and 29.07 (4.39) kg/m^2^, respectively. Table 1 presents the demographic information, lifestyle factors, and medical history of the study participants in both the case and control groups. Patients diagnosed with colorectal cancer (CRC) exhibited a significantly greater waist circumference (WC) in comparison to the control group (*p* < 0.05). Furthermore, the average consumption of vitamin D supplements and the HBI score among the case group was found to be considerably lower than that observed in the control group (*p*-value < 0.05). In contrast, for other characteristics and factors examined in the study, there were no significant differences identified between the patients in the case group and those in the control group.

**Table 1:** The demographic information, lifestyle factors, and medical history of the study participants in both the case and control groups.

Variables	CRC (*n* = 250)	Controls (*n* = 250)	*p*-value
Age (years)	48.91 ± 10.46	47.13 ± 10.08	0.274
Gender (female) *n* (%)	107 (43.1)	111 (44.6)	0.182
BMI (kg/m^2^)	29.61 ± 4.55	29.07 ± 4.39	0.354
WC (cm)	102.94 ± 10.77	97.04 ± 9.62	0.041
HBI score	56.98 (8.36)	60.92 (8.58)	<0.001
Marital status (married) *n* (%)	211 (84.4)	222 (88.8)	0.365
Physical activity (MET-h/wk)	35.18 ± 6.31	32.24 ± 5.94	0.198
Smoking (yes) *n* (%)	22 (8.8)	17 (6.8)	0.161
Family history of cancer (yes) *n* (%)	81 (32.4)	74 (29.6)	0.308
Vitamin D supplement (yes) *n* (%)	35 (14)	67 (26.8)	0.041
Calcium supplement (yes) *n* (%)	36 (14.4)	32 (12.8)	0.247

Table 2 presents a detailed overview of the mean food consumption patterns observed among the research participants, categorized into case and control groups. When making a comparison with the control group, it is evident that individuals diagnosed with CRC exhibited a higher intake of various macronutrients, including energy, carbohydrates, and fats. Furthermore, these subjects also consumed greater amounts of saturated fatty acids (SFA), cholesterol, additional carbohydrates, sodium, folate, iron, sugar-sweetened beverages, grains, and starches. Conversely, their consumption of protein, potassium, phosphorus, calcium, vitamin B12, and several micronutrients, including antioxidants such as zinc, magnesium, and vitamins E, C, and D, was notably lower. This difference in dietary intake was statistically significant, with a *p*-value of less than 0.05.

**Table 2:** Dietary intakes of study participants across case and control groups.

Variables	CRC (*n* = 250)	Controls (*n* = 250)	*p*-value^*^
Nutrients			
Energy (Kcal/d)	2642.45 (803.02)	2327.1 (617.43)	<0.001
Carbohydrate (g/d)	384.54 (7.01)	339.54 (5.21)	0.001
Protein (g/d)	78.08 (1.49)	89.47 (1.56)	<0.001
Fat (g/d)	109.62 (2.41)	92.14 (2.14)	<0.001
SFA (g/d)	33.45 (11.04)	28.90 (10.63)	<0.001
MUFA (g/d)	32.29 (13.29)	38.03 (16.03)	<0.001
PUFA (g/d)	19.48 (9.35)	23.49 (12.29)	<0.001
Cholesterol (mg/d)	307.52 (126.55)	264.88 (119.27)	0.009
Fiber (g/d)	30.14 (14.28)	39.89 (18.58)	0.048
Sodium (mg/d)	4851.74 (1811.95)	4217.06 (1898.50)	0.027
Potassium (mg/d)	3623.23 (1214.29)	4199.22 (1291.12)	<0.001
Calcium (mg/d)	1210.79 (433.90)	1345.27 (459.76)	0.005
Magnesium (mg/d)	368.06 (119.89)	411.91 (133.15)	0.004
Zinc (mg/d)	10.76 (3.62)	12.15 (3.95)	0.001
Vitamin C (mg/d)	147.16 (89.15)	186.87 (78.89)	<0.001
Folate (mcg/d)	495.57 (168.28)	448.20 (163.07)	0.025
Vitamin B12 (mcg/d)	4.53 (2.87)	5.70 (3.53)	0.002
Vitamin E (mg/d)	16.64 (12.16)	24.59 (16.54)	<0.001
Vitamin D (mcg/d)	1.92 (3.44)	2.71 (3.06)	0.019
Food groups			
Vegetables (g/d)	269.83 (150.72)	341.12 (142.46)	<0.001
Fruits (g/d)	425.98 (242.42)	533.17 (267.48)	<0.001
Fish (g/d)	11.04 (17.87)	14.56 (16.47)	0.037
Nuts (g/d)	9.09 (10.52)	12.14 (11.94)	<0.001
Red and processed meat (g/d)	32.84 (24.63)	29.01 (20.13)	0.317
Dairy foods (g/d)	452.12 (303.21)	527.53 (324.58)	0.084
Sugar-sweetened beverages (g/d)	82.66 (91.20)	63.63 (74.74)	0.041
Grains and starches (g/d)	432.31 (216.50)	364.82 (194.09)	0.031

SFA, saturated fatty acid; MUFA, monounsaturated fatty acid; PUFA, polyunsaturated fatty acids.

*Obtained from independent sample *T*-Test.

The dietary intakes, anthropometric measurements, and various lifestyle characteristics of the participants involved in the study, categorized according to their HBI quartiles, are presented in detail in Table 3. Notably, individuals who fell within the highest quartile of the HBI exhibited significantly greater consumption levels of calories, carbohydrates, proteins, fats, fibers, salts, potassium, calcium, magnesium, zinc, as well as vitamins C, E, and B9, when compared to those situated in the lowest quartile. This observation was statistically significant, with a *p*-value of less than 0.05. Furthermore, there was a marked increase in both the HBI score and the level of physical activity among participants as they progressed through the index quartiles.

**Table 3:** Dietary intakes, anthropometric, and lifestyle characteristics of study participants across quartiles of HBI.

Variables	Quartiles of HBI				*p*-value^*^
	Q1	Q2	Q3	Q4	
Age, (y)	50.64 (11.45)	49.12 (9.53)	47.32 (9.86)	47.01 (10.64)	0.096
BMI (kg/m^2^)	30.25 (4.84)	30.18 (4.82)	28.24 (5.08)	28.11 (5.06)	0.005
Waist circumference (cm)	101.33 (11.67)	100.62 (10.40)	97.47 (12.40)	98.22 (12.36)	0.002
Physical activity (MET.h/wk)	31.76 (5.09)	33.22 (5.57)	32.53 (5.37)	34.64 (6.23)	0.004
HBI score	49.96 (2.54)	57.12 (0.99)	61.95 (2.46)	69.71 (2.91)	<0.001
Nutrients					
Energy (Kcal/d)	2384.34 (672.81)	2465.33 (536.50)	2785.06 (754.04)	2936.64 (839.70)	<0.001
Carbohydrate (g/d)	310.08 (83.64)	341.64 (87.22)	379.22 (101.59)	404.78 (120.36)	<0.001
Protein (g/d)	73.10 (21.35)	82.71 (17.56)	91.06 (25.60)	96.04 (28.38)	<0.001
Fat (g/d)	102.06 (48.07)	90.02 (30.31)	104.71 (39.69)	108.78 (40.62)	0.031
SFA (g/d)	32.23 (12.89)	29.52 (9.35)	32.16 (11.82)	34.29 (11.69)	0.135
MUFA (g/d)	30.35 (17.78)	26.01 (10.69)	28.85 (12.04)	31.47 (12.11)	0.351
PUFA (g/d)	23.12 (15.31)	19.74 (9.04)	22.23 (12.41)	23.98 (10.59)	0.217
Cholesterol (mg/d)	271.59 (148.16)	295.50 (165.02)	278.42 (97.20)	321.48 (163.35)	0.265
Fiber (g/d)	28.74 (13.24)	33.18 (13.59)	45.29 (20.72)	46.79 (22.91)	<0.001
Sodium (mg/d)	4024.84 (1858.10)	4274.78 (1730.25)	4882.33 (1827.03)	4768.84 (1883.0.94)	0.035
Potassium (mg/d)	3316.61 (984.07)	3761.32 (911.08)	4273.42 (1284.72)	4674.49 (1466.13)	<0.001
Calcium (mg/d)	1141.34 (446.77)	1235.60 (378.72)	1371.45 (474.45)	1482.71 (541.02)	<0.001
Magnesium (mg/d)	319.89 (87.12)	364.82 (108.12)	419.30 (138.65)	445.61 (139.64)	<0.001
Zinc (mg/d)	10.68 (3.58)	11.71 (2.70)	13.64 (4.19)	14.28 (4.58)	<0.001
Vitamin C (mg/d)	131.89 (67.77)	158.87 (62.61)	211.81 (89.09)	231.87 (93.55)	<0.001
Folate (mcg/d)	409.95 (116.79)	467.15 (171.47)	505.84 (159.60)	545.78 (194.32)	<0.001
Vitamin B12 (mcg/d)	5.42 (4.16)	5.25 (3.08)	6.41 (3.84)	6.84 (4.83)	<0.001
Vitamin E (mg/d)	19.64 (23.22)	17.05 (9.62)	21.14 (14.44)	21.85 (11.44)	0.113
Vitamin D (mcg/d)	1.35 (1.64)	2.04 (1.74)	4.03 (6.86)	2.54 (2.02)	<0.001
Food groups					
Vegetables (g/d)	239.20 (94.48)	275.25 (113.82)	334.83 (133.72)	351.06 (157.41)	<0.001
Fruits (g/d)	351.37 (199.86)	403.26 (189.53)	532.85 (247.79)	586.18 (261.65)	<0.001
Fish (g/d)	8.25 (9.20)	11.32 (8.82)	21.96 (35.96)	13.61 (11.08)	<0.001
Nuts (g/d)	7.45 (5.59)	8.61 (10.15)	14.34 (15.86)	13.58 (11.32)	<0.001
Red and processed meat (g/d)	29.25 (30.65)	30.45 (17.51)	35.18 (25.60)	30.06 (19.29)	0.325
Dairy foods (g/d)	445.88 (308.01)	468.5766 (282.66)	507.04 (362.37)	573.24 (342.37)	0.378
Sugar-sweetened beverages (g/d)	63.31 (78.68)	65.46 (78.57)	60.84 (57.97)	70.46 (65.65)	0.627
Grains and starches (g/d)	365.84 (179.73)	438.26 (252.28)	409.57 (176.80)	431.71 (202.40)	0.478

SFA, saturated fatty acid; MUFA, monounsaturated fatty acid; PUFA, polyunsaturated fatty acids.

^*^Obtained from ANOVA.

Table 4 displays the odds ratios (ORs) and 95% confidence intervals (CIs) for colorectal cancer (CRC) patients categorized by HBI. In the crude model, the highest quartile of HBI scores, when compared to the lowest quartile, showed a reduction in CRC odds for the population (OR: 0.28; 95% CI: 0.19–0.51). Additionally, after further adjustments for potential confounders in the final adjusted model, the decrease in CRC odds remained statistically significant (OR: 0.43; 95% CI: 0.25–0.76 for the population).

**Table 4:** The odds ratios (ORs) and 95% confidence intervals (CIs) for colorectal cancer (CRC) patients categorized by HBI.

CRC	Quartiles of HBI*				*p*-value
	Q1	Q2	Q3	Q4	
Crude model	1.00 (Ref)	0.49 (0.27–0.85)	0.22 (0.11–0.45)	0.28 (0.19–0.51)	<0.001
Model 1*	1.00 (Ref)	0.51 (0.31–0.91)	0.25 (0.15–0.48)	0.32 (0.18–0.55)	<0.001
Model 2^†^	1.00 (Ref)	0.56 (0.31–0.95)	0.34 (0.18–0.57)	0.39 (0.22–0.69)	<0.001
Model 3^#^	1.00 (Ref)	0.59 (0.32–1.08)	0.31 (0.17–0.57)	0.43 (0.25–0.76)	0.001

*Binary logistic regression was used to obtain OR and 95% CI.

Model 1: adjusted for age and sex.

^†^Model 2: Model 1 + BMI, waist circumference, physical activity, and energy intake.

^#^Model 3: Model 2+ smoking, marital status, family history of cancer, hormone replacement therapy, calcium supplement, vitamin D supplementation, dairy, fruit, and vegetable intake.

## Discussion

The aim of this hospital-based study was to investigate the relationship between consumption of healthy beverages and risk of colorectal cancer (CRC). The study included patients with CRC and control participants chosen from individuals with similar demographic characteristics. An anthropometric measurement and physical activity level measurement was performed according to standardized methods, and dietary intake was assessed using a food frequency questionnaire. The HBI was used to evaluate the quality of beverage consumption and the possibility that healthier beverage choices may reduce CRC risk. Those with higher adherence to healthy beverage consumption patterns were less likely to develop colorectal cancer than those with less healthy drinking habits, the results showed. The role of diet and lifestyle factors in preventing disease, in particular colorectal cancer, is stressed in this study.

To gain insight into the long-term effects of healthy beverage consumption on colorectal cancer (CRC) risk and whether behavioral interventions can change beverage consumption patterns, researchers leverage both longitudinal studies and randomized controlled trials (RCTs) (13). Evidence for such an effect is obtained from long-term observational studies that demonstrate that consumption of beverages containing green tea, coffee, and some fruit juices is associated with reduced CRC risk because of their anti-inflammatory and antioxidant properties (14). For example, the Nurses’ Health Study and the Health Professionals Follow-up Study, as these have over the decades tracked health behaviors, indicate that there is a slight but real connection between regular coffee consumption and some reduction in CRC risk (15). These studies point to the influence of long-term dietary habits on cancer risk.

Conversely, beverages low in nutrients and high in sugar, such as sugar-sweetened sodas and certain fruit-flavored drinks, have been associated with an increased risk of CRC. High consumption of these beverages can contribute to systemic inflammation, insulin resistance, and obesity, all of which are established risk factors for CRC (8, 16). For instance, a longitudinal study by Hur et al. (16) found that sugar-sweetened beverage intake during adulthood and adolescence was linked to an elevated risk of early-onset CRC in women, potentially due to the disruption of glucose metabolism and promotion of pro-inflammatory pathways. Similarly, Llaha et al. (17) reported that sugary beverages, including sodas and sweetened fruit juices, are associated with increased cancer risk, likely through mechanisms such as increased visceral adiposity and oxidative stress. These findings suggest that replacing nutrient-poor, high-sugar beverages with healthier alternatives, such as water or unsweetened teas, could mitigate CRC risk by reducing metabolic and inflammatory stressors.

The RCTs and behavioral interventions evaluate the effectiveness of particular change strategies for inducing change in beverage consumption patterns. For instance, some success has been achieved in changing long-term consumption habits through interventions that include educational components about the benefits of certain beverages and personalized feedback (18). Follow-ups are often used in these studies to find out if changes in beverage consumption have a lasting impact on health outcomes (e.g., CRC risk). Validation of the protective effects of beverage consumption against CRC and the development of effective public health strategies to promote long-term healthy beverage choices are these research efforts (19).

Dietary and lifestyle factors which influence colorectal cancer (CRC) risk have been extensively researched. Bahrami et al. (20) reported that diets rich in calcium, vitamin C, riboflavin, folate, and fiber are protective against CRC and adenoma, while Fereidooni et al. (21) found that a healthy dietary pattern is associated with decreased CRC risk and a Western dietary pattern increases it. Aleksandrova et al. (22) showed that a lifestyle index composed of five modifiable factors decreases risk of CRC, Reedy et al. (23), as well as Steck et al. (24), linked adherence to dietary guidelines and anti-inflammatory diets to lower CRC risk, Tabung et al. (25), as well as Magalhães et al. (26), confirmed the protective role of fruit and vegetable rich diets and Mediterranean dietary patterns.

On the other hand, Vieira et al. (27) found that red/processed meats and alcohol increase CRC risk, but milk and whole grains are protective. In women, sugar-sweetened beverages in adulthood and adolescence raise the risk of early-onset CRC, Hur et al. (16) found. Huxley et al. (28) outlined the complete picture of dietary and lifestyle risk factors for CRC outcomes. Other studies include Jafari Nasab et al. (29) who found that higher Healthy Eating Index scores are associated with a reduced CRC risk; Hang et al. (30) who showed the combined effect of lifestyle factors and comorbidities on CRC risk; and Tabung et al. (25) who found that proinflammatory diets increase CRC risk, especially in postmenopausal women.

While Duffey and Davy (12) and Jahanbazi et al. (31) found that the HBI was associated with lower cardiometabolic risk, there was no direct association with CRC. Mahmoodi et al. (32) and Rasaei et al. (33) examined the HBI association with sarcopenia and obesity, rather than CRC. HBI’s inverse relationship with non-alcoholic fatty liver disease was investigated by Sadafi et al. (34).

There are many studies that have shown that dietary patterns and some beverages are associated with colorectal cancer risk. Some examples are that of Llaha et al. (17) who found that sugary beverage consumption (such as sodas or fruit juices) is related to an increased risk for some cancers. Vieira et al. (27) also reviewed that high intake of red and processed meats and alcohol increases the risk of colorectal cancer, but milk and whole grains have a protective effect. Like Huang et al. (35) found no significant association between tea consumption and colorectal cancer risk. According to Je et al. (36), high coffee consumption has no significant effects on colorectal cancer incidence, and according to a meta-analysis by Giovannucci (37), coffee consumption might reduce risk for colorectal cancer, but the results are not definitive. An independent study conducted by Akter et al. (38) has shown that a higher or reduced coffee consumption is not associated with increased, nor decreased colorectal cancer risk in the Japanese population. There, research by Schwingshackl et al. (39) showed that a high intake of whole grains, vegetables, fruits, and dairy is associated with a lower risk of colorectal cancer and high intake of red and processed meats with a higher risk.

Several biological mechanisms have been identified as being associated with a reduced risk of colorectal cancer (CRC) through consumption of healthy beverages, such as green tea, coffee, and certain fruit juices. Importantly, these beverages are rich in antioxidants, polyphenols, and other bioactive compounds, which are well known to play important roles in preventing cancer (40). An example is green tea with rich content in catechins, especially epigallocatechin-3 gallate (EGCG). It is shown that EGCG can induce apoptosis (programmed cell death) and arrest cells in the cell cycle, which research indicates inhibits the growth of colorectal cancer cells. In addition, it also has anti-inflammatory properties that may lower the chronic inflammation associated with increased cancer risk (41). By reducing oxidation, caffeine and other antioxidants present in coffee may protect the colon from digestion of carcinogens and the development of oxidative stress as seen in the colonic mucosa (42). Coffee also contains anti-carcinogenic chlorogenic acids that modulate the expression of genes related to tumor necrosis and cell proliferation (43). Besides, some fruit juices such as those from berries and citrus fruits are high in vitamin C, flavonoids, and other phytochemicals. They have been shown to inhibit the formation of cancer cells by means of their antioxidant activity and their ability to modulate detoxification enzyme functions (44). In contrast, high-sugar, low-nutrient beverages may exacerbate CRC risk by promoting chronic inflammation and insulin resistance. For example, excessive sugar intake can lead to elevated blood glucose levels, which may stimulate insulin-like growth factor (IGF-1) signaling pathways, promoting cell proliferation and inhibiting apoptosis in colorectal tissue (8). Additionally, high-sugar beverages contribute to weight gain and visceral fat accumulation, which are linked to increased CRC risk through inflammatory cytokine production and altered gut microbiota (16, 17). The consumption of these healthy beverages provides a potential dietary strategy for the reduction of the risk of colorectal cancer by multiple protective mechanisms: antioxidant activity, anti-inflammatory effects, and stimulation of cell growth and apoptosis, while avoiding high-sugar beverages can prevent adverse metabolic effects (45).

This study concludes that there appears to be an association between good health beverage consumption (as measured by the HBI) and reduced risk of CRC. This suggests that beverage consumption might be modified as a preventive strategy. Reducing risk may also be promoted by encouraging healthier beverage choices, such as water, unsweetened beverages, and low-fat milk, while discouraging the consumption of sugar-sweetened beverages. Nevertheless, this study has idiosyncratic limitations, and additional research is needed to verify these findings and reveal the mechanistic basis for them. These results can inform public health professionals and clinicians on how to provide dietary advice to reduce CRC risk. CRC prevention requires important public education about the importance of healthy beverage choices, as well as the development and implementation of interventional programs to modify beverage consumption patterns.

The hospital-based case-control study increases the risk for selection bias. Assessment of beverage intake using a food frequency questionnaire is subject to recall bias. Limitations to generalizability of the findings exist due to study focus on a Saudi population. Although some were controlled for confounders, others may not have been. Additionally, the inability to fully assess the maximum HBI score was also present because some beverage types were not present in the study population. Limitations to these studies could be overcome in future studies with more precise measures of beverage intake, such as 24-h dietary recalls. To increase generalizability, longitudinal studies need to include larger, more diverse populations. One limitation of our study is that beverage intake was assessed based on consumption over the past year. Given the long latency period of colorectal cancer, this timeframe may not fully capture long-term dietary exposures that contribute to disease development. However, adult dietary patterns are generally stable over time, and recent intake may still play a role in cancer progression or influence risk in the late stages of carcinogenesis. Another limitation of the present study is the modification of the original HBI to better fit the beverage consumption patterns and available data in our population. Although this adaptation increases the contextual relevance, it may reduce the comparability of our findings with studies using the original HBI scoring system, potentially limiting the generalizability of our results across different populations.

## Conclusion

The findings suggest that adherence to a healthier beverage consumption pattern, as measured by the HBI, is significantly associated with a reduced risk of colorectal cancer (CRC). These findings suggest that there is the potential to alter beverage choices, for example by increasing water and unsweetened tea intake and nutrient dense beverages intake and reducing sugary and alcoholic beverage intake, as a potent CRC prevention strategy. While we consistently observed protective effects of the HBI, even after adjusting for confounding factors, further research with more diverse populations and more sophisticated assessment tools is needed to confirm these findings and to elucidate the underlying biological mechanisms. These insights are important to be used to develop dietary strategies targeted at reducing CRC risk and healthier lifestyles, and for designing public health policies.

## Data Availability

The raw data supporting the conclusions of this article will be made available by the authors, without undue reservation.
